# Carcass Traits and Meat Quality of Surgically Castrated and Immunocastrated Pigs at Two Slaughter Weights

**DOI:** 10.3390/ani15192846

**Published:** 2025-09-29

**Authors:** Dmytro V. Zhdanov, Oleksandr H. Mykhalko, Mykola H. Povod, Galia Zamaratskaia

**Affiliations:** 1 Department of Feed Technology and Animal Feeding, Sumy National Agrarian University, 40021 Sumy, Ukraine; number5070@ukr.net (D.V.Z.); snau.cz@ukr.net (O.H.M.); nic.pov@ukr.net (M.H.P.); 2 Department of Molecular Sciences, Swedish University of Agricultural Sciences (SLU), 750 07 Uppsala, Sweden

**Keywords:** surgical castration, immunocastration, pigs, carcass traits, meat quality, slaughter weight, muscle pH

## Abstract

**Simple Summary:**

This study compares two methods of pig castration, surgical castration and immunocastration, focusing on their effects on growth, carcass traits, and meat quality at two slaughter weights. Surgical castration has been widely used to prevent unpleasant odours and aggressive behaviour in male pigs but raises animal welfare concerns and leads to fattier carcasses. Immunocastration is a vaccine-based alternative that improves welfare by avoiding surgery and is widely recognized to reduce the risk of boar taint and aggressive behaviour. Our results show that immunocastrated male and female pigs have carcass and meat quality traits generally between those of surgically castrated males and intact females. Differences in fat content, meat pH, and colour were influenced by castration method and sex. In addition to surgically and immunocastrated males, the study included uncastrated and immunocastrated females to provide novel comparative data relevant for production systems finishing both sexes. While data on females remain limited, including these groups allows a broader evaluation of immunocastration effects. The findings support immunocastration as an approach that maintains pork quality, providing valuable information for pig producers and consumers.

**Abstract:**

Surgical castration of male piglets is a common practice to prevent boar taint and reduce aggressive behaviour. However, it raises welfare concerns and alters carcass fat deposition. Immunocastration, a vaccine-based alternative targeting gonadotropin-releasing hormone (GnRH), mitigates these welfare issues. This study evaluated carcass traits and meat quality in surgically and immunocastrated pigs slaughtered at two weight classes (approximately 116 kg and 136 kg). We compared growth performance, carcass composition, fat quality, and key meat quality indicators among surgically castrated males, immunocastrated males, and immunocastrated females. Inclusion of uncastrated and immunocastrated females provides novel comparative data for mixed-sex production systems, where such information is scarce. This broader evaluation helps fill current gaps in knowledge about immunocastration effects in female pigs. Surgically castrated males showed higher backfat thickness and fat content, particularly at the heavier weight, while immunocastrated pigs exhibited intermediate traits. Ultimate pH, colour, marbling, water-holding capacity, and moisture loss varied with castration method, sex, and slaughter weight, though many differences were subtle. The findings confirm that immunocastration offers a favourable balance between animal welfare and production traits, producing pork quality comparable to surgical castration. These results provide valuable insights for optimizing pork production systems, balancing welfare, efficiency, and meat quality.

## 1. Introduction

Surgical castration of male piglets has been a standard husbandry practice worldwide for decades. The procedure aims to prevent the accumulation of boar taint compounds (mainly androstenone and skatole) in adipose tissue and to reduce male aggressive and sexual behaviours during the finishing period [1,2]. However, routine surgical castration performed without adequate anaesthesia or analgesia causes significant acute pain and measurable welfare costs to piglets [3]. This practice is increasingly subject to regulatory restrictions, as well as rising ethical concerns from retailers and consumers [4]. At the same time, castration increases feed conversion costs and results in fatter carcasses with different fat composition and technological properties compared with uncastrated males (entire males), which typically produce leaner carcasses and distinct meat quality attributes. Thus, there is growing interest in alternatives that avoid the welfare costs of surgical castration while effectively controlling boar taint and maintaining acceptable production and meat-quality traits.

Immunocastration, active immunization against gonadotropin-releasing hormone (GnRH), is one such alternative that has been the subject of extensive experimental and field evaluation [5,6,7,8,9]. The technique uses a vaccine (commercial examples include Improvac^®^ produced by Zoetis) administered in a two-dose schedule to induce neutralizing antibodies against endogenous GnRH. Following the booster (second) dose, pituitary secretion of luteinizing hormone (LH) and follicle-stimulating hormone (FSH) is reduced, testicular steroidogenesis and androgen production fall, and testicular function regresses [7]. The resulting endocrine state mimics the hypogonadal status of surgical castrates, and the levels of testosterone and testicular steroids decline. The levels of skatole are also reduced by immunocastration by lowering testicular steroid hormones, which normally inhibit hepatic cytochrome P450 enzymes responsible for skatole metabolism. When androgen levels fall after vaccination, hepatic clearance of skatole increases, leading to rapid declines in tissue levels [10,11]. The physiological basis and timeline of these changes have been repeatedly documented in controlled trials.

The efficacy of GnRH-based immunocastration in eliminating boar taint and improving handling and welfare is supported by early large-scale trials and subsequent meta-analyses and reviews [1,12,13]. Initial field and experimental studies showed that vaccinated boars had substantially reduced concentrations of androstenone and skatole at slaughter and often displayed improved growth performance compared with surgical castrates while avoiding the pain and welfare costs associated with surgery [7,14]. Meta-analytic syntheses confirm that immunocastration produces large, consistent effects on reproductive organ size and on boar taint compounds and that the typical production pattern is “boar-like” performance up to the booster immunization followed by a metabolic shift toward a castrate-like profile [15]. Consumer panels, sensory trials, and chemical analyses agree that immunocastrated pork has the same quality as pork produced by surgical castrates, with no significant differences found in pH, drip loss, marbling, tenderness, or colour [16,17]. Immunocastrated pigs often display intermediate intramuscular fat (IMF) levels and meat tenderness compared to entire male pigs and surgically castrated pigs [18,19]. Żakowska-Biemans et al. [20] found that dry-cured pancetta and salami from immunocastrated and surgically castrated pigs had very similar sensory profiles and liking scores, while the products from entire male pigs were more often associated with boar taint odours and firmer, leaner texture due to lower fat content. Importantly, consumer acceptance was influenced not only by boar taint but also by fat content and texture. These results establish immunocastration as an effective tool to control boar taint while improving animal welfare relative to conventional castration without pain control [1].

Immunocastrated pigs typically behave and grow like entire males until the antibody response suppresses testicular function. After that point, feed intake often increases and lean-to-fat deposition shifts rapidly, creating carcasses and fat with intermediate traits between entire males and early surgical castrates [12,14]. The magnitude and direction of these changes depend on vaccination schedule, genotype, feeding regime and the time from booster to slaughter [7,21]. Meta-analyses and recent reviews detail these performance and carcass trade-offs and show that, on average, immunocastration offers a favourable balance between welfare gains and production costs when correctly implemented [1,15].

From the meat quality and processing perspective, tissue composition differences between entire males, immunocastrates, and surgical castrates are well described [22]. Entire males typically yield leaner carcasses with higher proportions of polyunsaturated fatty acids and lower intramuscular fat. These properties are advantageous for fresh lean cuts but might be problematic for processing (dry-cured products) where fat firmness, oxidative stability, and specific sensory attributes are important [8,21]. Immunocastration tends to produce carcass and fat quality intermediate between entire males and surgical castrates, often acceptable for many fresh-meat products but requiring attention for certain processed products [8,23]. Studies comparing immunocastrates, entire males, and surgical castrates across product types show that appropriate scheduling and minor processing adaptations (formulation, antioxidants, smoking or masking strategies) can mitigate many of the quality issues while preserving animal welfare benefits [21,23].

Most immunocastration studies have focused on male pigs with a wealth of data on growth performance, boar taint control, carcass traits, and welfare outcomes [12,14,18]. Studies on female pigs are fewer and often preliminary, though they nonetheless indicate that immunocastration via GnRH vaccination effectively suppresses reproductive function in gilts without compromising growth or meat quality. For example, Zeng et al. [24] demonstrated that active immunization in Chinese crossbred female pigs reduced serum LH and inhibin A levels, and markedly decreased ovarian and uterine weights while showing no significant adverse effects on growth. Xue et al. [25] likewise found that immunocastrated SuHuai gilts exhibited suppressed ovarian development, reduced progesterone concentrations, and no detrimental effects on carcass or meat-quality traits compared with controls. More data from culled sows also show that reproductive tract regression occurs without negative impacts on carcass or meat quality [26].

Slaughter weight is an important determinant of carcass yield, fat deposition, and meat quality, and is therefore a critical variable when comparing different production systems and castration strategies. Increasing slaughter weight generally improves carcass yield and lean meat percentage but it is often associated with higher backfat thickness and IMF content, which can positively affect juiciness and flavour but may reduce carcass grading and processing suitability [27]. In immunocastrated pigs, the timing of vaccination relative to slaughter weight is especially relevant because the duration of the “boar-like” growth phase before the second dose determines lean deposition. At the same time, heavier slaughter weights may amplify fat deposition shifts after gonadal suppression [28]. Thus, evaluating the effects of castration method at different slaughter weights is important to understand potential interactions between gonadal status, growth performance, and meat-quality outcomes under commercial conditions.

This study aimed to evaluate the effects of surgical castration and immunocastration on carcass traits and meat quality parameters in pigs slaughtered at two different weight classes. Specifically, we compared growth performance, carcass composition, fat quality, and key meat quality indicators between surgically castrated males, immunocastrated males, uncastrated females, and immunocastrated females. While some production systems, such as those in the UK, raise entire (uncastrated) males to avoid castration, this study focused on comparing surgical and immunocastration due to their widespread use globally and to assess immunocastration as a welfare-friendly alternative.

## 2. Materials and Methods

### 2.1. Animals

A total of 810 pigs (sows of the cross Landrace × Large White, mated with boars of the PIC-337 terminal line) were included in the study. They were categorized into four experimental groups combining sex and castration method (surgically castrated male pig, immunocastrated male pigs, uncastrated female pigs, and immunocastrated female pigs).

On days 2–3 of life, four piglets (two females and two males) were selected from full litters of sows. Each animal was marked with coloured numbered ear clips according to the research protocol, with each group assigned its own colour and numbering. All males of the control group underwent surgical castration, while the remaining animals were raised together without alterations. At weaning (day 28), piglets were individually weighed, additionally tagged with numbered ear tags corresponding to their original clip numbers and colours, and divided into four groups according to sex and castration method. They were placed in pens for rearing, with 55–60 animals per pen, providing a floor space of 0.35 m^2^ per weaner. Pens were fully slatted, and animals were fed liquid diets at a feeding space allowance of 0.09 m per head. At day 77, pigs were again individually weighed, and the final experimental groups were formed and transferred to the fattening stage. An initial live weight of pigs was 28.2 ± 4.11 kg (mean and standard deviation).

During finishing, animals were kept under standard industrial conditions in group pens of 50 pigs, on fully slatted floors with a space allowance of 0.75 m^2^ per animal, and with an automated feeding system. Feeding was provided 10–14 times daily in the form of liquid feed mixtures with a dry-to-liquid ratio of 1:2.7–3.0. Feed intake was recorded by the feeding system processor for each pen and averaged 2.49 kg in surgically castrated males, 2.48 kg in immunocastrated males, 2.36 kg in entire females, and 2.39 kg in immunocastrated females.

All pigs received standard diets formulated to meet nutrient requirements for each growth stage. Diets were offered ad libitum and differed by growth phase: grower (30–60 kg), finisher I (60–90 kg), and finisher II (90–130 kg). The detailed composition of each ration is provided in Table 1. Major ingredients included wheat, maize (corn), soybean meal, sunflower meal, and supplementary minerals and amino acids. No antibiotics were included except tiamulin in the grower feed at 0.10 g/kg, and a standardized premix provided vitamins, minerals, and trace nutrients throughout the finishing stages. Additives, including lysine, methionine, threonine, and others, were supplemented to achieve targeted amino acid profiles for optimal growth and carcass quality.

The Improvac^®^ (Zoetis Inc., Malvern, PA, USA) vaccination protocol was applied according to the manufacturer’s instructions (two doses 4 weeks apart, with slaughter occurring at least 5 weeks after the second vaccination). On day 112, all entire males and half of the females were vaccinated with Improvac^®^ (2 mL per animal). The second dose was administered at 140 days of age with the same vaccine and dose. Both groups were vaccinated at the same time points. Half of the females received no intervention. Slaughter occurred simultaneously for all groups. Differences in slaughter weight reflected individual growth rates.

At day 177, when the pigs reached an average live weight of 120 kg, the pigs were weighed again, and the average daily gain was calculated. Initially, four experimental groups of 20 pigs each (a total of 80 animals) were formed. They were weighed, and each group was subdivided into two subgroups with preslaughter live weights of 115.8 ± 2.61 kg (mean ± standard deviation) and 136.0 ± 1.52 kg, respectively. For clarity, throughout the manuscript, the first subgroup will be referred to as the “lighter pigs” and the second as the “heavier” pigs. The slaughter weights of approximately 116 kg and 136 kg correspond to typical commercial finishing weights considered relatively heavy in pork production systems. Including both weights allows evaluation of how castration methods influence carcass traits and meat quality across these common heavy finishing stages, where fat deposition and meat characteristics can be markedly affected. During unloading, one pig from the group of heavier surgical castrates jumped out of the unloading device, sustained an injury, and was subjected to sanitary slaughter on welfare grounds. Consequently, this pig was removed from the study, and the final number of pigs included in the analysis was 79.

Live weight shrink was determined as the difference between the pig’s live weight measured at the farm before transport and the live weight recorded at the slaughterhouse before slaughter. Specifically, live weight shrink was calculated by subtracting the slaughter weight from the farm weight for each animal. This variable represents the reduction in body weight primarily due to fasting, dehydration, and stress during transport.

Animals were slaughtered at Hlobyne Meat Processing Plant LLC (Hlobyne, Kremenchuk District, Poltava Region, Ukraine), where carcass composition and meat quality were subsequently analyzed in the plant’s laboratory.

### 2.2. Sample Collection and Carcass Measurements

After slaughter, samples of the longissimus dorsi muscle were collected from the region between the 9th and 12th thoracic vertebrae. Hot carcass weights were recorded immediately post-slaughter (hot carcass weight) and again after chilling (chilled carcass weight). After chilling, the loin eye area was determined as the cross-sectional area of the longissimus dorsi muscle at the level between the first and second lumbar vertebrae. The muscle contour was traced onto tracing paper, after which the area was measured using the AutoCAD 2023 (Autodesk Inc., San Francisco, CA, USA, Education License) software. The muscle was fully dissected during carcass deboning and weighed with an accuracy of 0.01 kg. The weight of the posterior third of the half-carcass was measured on chilled carcasses following a transverse cut between the last and penultimate lumbar vertebrae and expressed in kilograms. Backfat thickness was measured on the chilled carcass above the spinous processes between the sixth and seventh thoracic vertebrae, excluding the skin, using a standard ruler, and expressed in millimeters.

### 2.3. Moisture Loss and Water-Holding Capacity

Moisture loss was assessed by suspending muscle samples in sealed plastic bags at 4 °C and reweighing after 48 h. The percentage of juice loss was calculated as the difference in sample weight before and after storage relative to the initial weight, multiplied by 100.

Water-holding capacity (WHC) was determined on the third day post-slaughter using a modified press method [29]. Approximately 500 mg of ground meat per sample was pressed between plexiglass plates under 1 kg weight for 10 min. The wet spot area was outlined and measured after drying. The muscle contour was traced onto tracing paper, and the area was calculated using AutoCAD software. Bound water content (%) was calculated based on the spot area and initial moisture according to established formulas.

### 2.4. pH Measurements

Post-mortem muscle pH was measured with a calibrated Testo 205 pH meter at 15 min, 24 h, and 48 h after slaughter.

### 2.5. Colour and Marbling

Muscle colour and marbling scores were assessed visually by a trained panel of five evaluators using the colour scale and a 6-point (colour) or 5-point (marbling) scale [30]. Panellists independently scored cross-sectional cuts, and average values were calculated.

### 2.6. Chemical Composition

Moisture, protein, fat, and collagen contents were determined using a FOSS FoodScan Meat analyser (FOSS, Hilleroed, Denmark). Measurements were conducted according to the manufacturer’s official calibrations and sample preparation procedures [31]. Fresh muscle homogenates were analyzed within 30 min of preparation under controlled room temperature.

### 2.7. Statistical Analysis

Data were analyzed using SAS software (version 9.4; SAS Institute, Cary, NC, USA). First, stratified analyses by weight class (lighter or heavier pigs) were performed to account for the confounding effect of weight on growth- and carcass-related variables using General Linear Models. Least Squares Means (LSMeans) for the groups were estimated and compared with Tukey–Kramer adjustment for multiple comparisons. Due to partial confounding of treatment combinations with sex and weight groupings, a combined factor representing each unique treatment–sex–weight combination was used as a categorical fixed effect. Initial live weight was included as a covariate to adjust the models for pre-slaughter body weight differences. Then, we further analysed the effect of slaughter weight category on selected variables within each treatment group (surgically castrated males, immunocastrated males, uncastrated females, and immunocastrated females). The variables examined included backfat thickness, loin muscle cross-sectional area and weight, key meat quality traits (colour, marbling, water-holding capacity, moisture loss), post-mortem pH measured at multiple time points, and proximate meat composition (protein, fat, moisture, and collagen). These variables were chosen because they represent critical indicators of carcass composition and meat quality known to be influenced by slaughter weight and of practical relevance to production and processing outcomes. Statistical comparisons between the lighter and heavier weight categories within each treatment group were conducted using General Linear Models with Tukey–Kramer adjustment for multiple comparisons. Differences were considered significant at *p* < 0.05.

## 3. Results

### 3.1. Growth Performance

**Figure 1:**
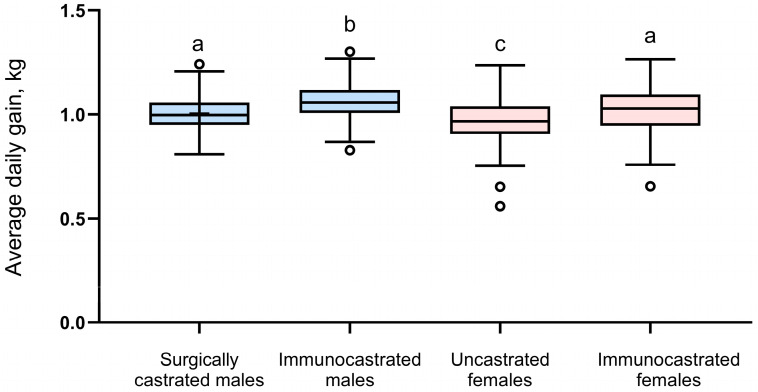
Distribution of average daily gain (kg) across four treatment groups. Boxes represent the interquartile range (IQR), with the median shown as a horizontal line. Whiskers (error bars) extend to the smallest and largest observations that are not considered outliers; circles indicate outliers beyond this range. Blue boxes represent males and pink boxes represent females. Overall differences among groups were significant (ANOVA, *p* < 0.001). Groups not sharing the same superscript letter differ significantly (*p* < 0.05).

ANOVA analysis revealed a highly significant effect of treatment on average daily gain (Figure 1; *p* < 0.0001). Subsequent Tukey–Kramer adjusted pairwise comparisons showed that immunocastrated males (1.06 kg) exhibited significantly greater average daily gain than all other groups, whereas uncastrated females (0.97 kg) had the lowest values. Surgically castrated males (1.00 kg) and immunocastrated females (1.02 kg) did not differ from each other.

In the lighter weight group, live weight shrink differed significantly among surgically castrated males, immunocastrated males, uncastrated females, and immunocastrated females (*p* < 0.001), with immunocastrated males showing the greatest shrinkage and immunocastrated females the least (Table 2). Hot carcass weight and chilled carcass weight did not differ significantly between groups (*p* = 0.348 and *p* = 0.440, respectively). Hind third weight showed a significant difference (*p* = 0.001), with surgically castrated males having lower values compared to uncastrated and immunocastrated females.

In the heavier weight group, live weight shrink did not differ significantly among treatments (*p* = 0.086; Table 3). Significant differences were observed in hot carcass weight and chilled carcass weight (both *p* < 0.001), with surgically castrated males exhibiting higher values compared to all other groups. For chilled carcass weight, immunocastrated males were significantly lighter than uncastrated and immunocastrated females. Hind third weight also differed significantly (*p* < 0.001), with uncastrated females having the highest weights and surgically castrated males having the lowest.

### 3.2. Effect of Castration Method Within Live Weight Categories

Post-mortem pH profiles showed significant variation across treatment groups and live weight categories (Table 4). At 48 h post-mortem, pH values differed significantly (*p* < 0.001). In lighter pigs, immunocastrated males and females exhibited lower pH compared to surgically castrated males and uncastrated females. In heavier pigs, surgically castrated males had a higher pH than all other groups. At 6 days post-mortem in heavier pigs, surgically castrated males retained a significantly higher pH than immunocastrated males, with female groups showing intermediate values (*p* = 0.024). Furthermore, in the ham muscle, uncastrated females had the highest pH. This was significantly higher than all other groups in heavier pigs and higher than surgical and immunocastrated males (with immunocastrated females being intermediate) in lighter pigs. Early post-mortem measurements at 45 min and 24 h showed no significant treatment effects (*p* > 0.05).

Backfat thickness was greater in surgically castrated males compared to immunocastrated males in heavier pigs (*p* = 0.028, Table 5). Muscle colour scores differed significantly in the lighter weight group (*p* = 0.012), being higher in uncastrated female pigs compared to surgically and immunocastrated males, with immunocastrated females being intermediate. Other attributes, such as loin area, weight, marbling, WHC, and moisture loss, showed less pronounced or nonsignificant differences across groups.

The proximate composition of meat differed among treatment groups (Table 6). In pigs of lighter weight, protein content varied significantly across groups (*p* = 0.047), with immunocastrated males and uncastrated females exhibiting higher protein percentages compared to surgically castrated males. Fat, moisture, and collagen contents did not differ significantly in this weight category. For heavier pigs, fat content showed significant differences (*p* = 0.036); surgically castrated males had higher fat levels compared to immunocastrated males, while uncastrated and immunocastrated females had intermediate fat values. Protein, moisture, and collagen percentages were similar across groups in the heavier weight category.

### 3.3. Comparison Between Live Weight Categories Within Castration Groups

The effect of slaughter weight on pH was not consistent across all treatments, but was significant in uncastrated female pigs (Table 7). In this group, a higher slaughter weight was associated with a significant reduction in 48 h, pH (5.8 vs. 5.5) and a significant increase in ham muscle pH (5.8 vs. 6.0). In contrast, no significant differences in pH between the two slaughter weights were observed for surgically castrated males, immunocastrated males, or immunocastrated females at any measured time point (*p* > 0.05).

The impact on carcass and meat quality traits was also treatment-specific. Immunocastrated males and both female groups exhibited a significant increase in loin weight with higher slaughter weight (*p* < 0.05). Furthermore, immunocastrated and uncastrated females showed a significant increase in longissimus dorsi cross-sectional area (*p* < 0.05). A darker meat colour (higher score) was found in heavier immunocastrated males (*p* = 0.005). In surgically castrated males, the higher slaughter weight resulted in a significant reduction in chilling loss (shrinkage, *p* < 0.001) and a significant increase in hot and cold carcass weight (*p* < 0.001). The proximate composition of the meat was largely unaffected by slaughter weight across all treatment groups (*p* > 0.05).

## 4. Discussion

The effective management of carcass traits and meat quality remains a central focus in pig production. This study investigated the impact of castration methods and slaughter weights on key carcass and meat quality traits in pigs. The results showed how castration method and slaughter weight affect growth, carcass composition, and pork quality under commercial conditions.

Female pigs are castrated (spayed) or immunocastrated mainly to prevent unwanted pregnancies and to manage sexual behaviour in production systems, especially where females might encounter males (including wild boars) during free-range rearing. While male pig castration predominantly targets boar taint and aggressive behaviours, spaying and immunocastration in females aim to manage breeding cycles and avoid sanitary or management complications that could arise from uncontrolled mating [32].

The effects of increasing slaughter weight (116–136 kg) were not consistent across treatments but depended on sex and castration status. While the higher weight universally increased final live weight, its impact on carcass and meat quality parameters was highly treatment-specific. Live weight shrinkage during transport and lairage is a well-documented phenomenon primarily caused by fasting, dehydration, and stress, which can result in significant weight loss before slaughter [33]. The greater live weight shrink observed in immunocastrated males in our study might be explained by physiological and metabolic differences associated with immunocastration and sex. Rapid reduction in testicular steroids after vaccination shifts metabolism from a boar-like to a castrate-like profile. This transition can lead to increased voluntary feed intake and lipid deposition in males, which may also involve changes in water retention, gut fill, and stress responsiveness during transport, contributing to greater weight fluctuations [34]. In immunocastrated males, this elevated shrinkage likely reflects the marked physiological transition that occurs after the second vaccination [12]. Following immunocastration, feed intake typically rises as sex steroid production and steroid feedback decline [15]. This leads to greater gut fill before fasting and subsequently more weight loss during the transport and lairage period. Moreover, lipid deposition accelerates while protein accretion slows, altering water turnover and body composition. Behavioural changes also play a role [13]. Immediately after vaccination, pigs may still exhibit male-like behaviour and stress responsiveness, as the endocrine suppression develops gradually. This can contribute to greater handling difficulty and stress during transport compared with surgically castrated animals. These physiological and behavioural changes not only influence carcass and meat quality traits but might also affect consumer acceptability by modulating factors such as meat texture, juiciness, and visual appeal.

The ultimate pH (48 h) exhibited a complex interaction between sex and weight. Immunocastrated pigs of both sexes consistently showed lower ultimate pH. However, the response of uncastrated females was weight-dependent; they maintained a higher pH at lighter weights but a lower pH at heavier weights, similar to immunocastrated pigs. Conversely, in the ham muscle, uncastrated females had the highest pH regardless of weight category. These findings align with previous research indicating that the metabolic response, particularly glycogen stores and post-mortem glycolysis, is intrinsically linked to sexual maturity and hormonal status [15]. The weight-dependent effect in uncastrated females particularly supports the concept that sex interacts with weight for pH and related traits, as reported in a meta-analysis by Trefan et al. [35]. This interaction might reflect distinct shifts in muscle metabolism and fatness trajectories as females reach heavier weights, leading to different glycolytic potential in different muscles (loin vs. ham). Muscle glycogen serves as the key substrate for post-mortem glycolysis, producing lactic acid that lowers pH from physiological levels near 7.4 to an ultimate pH of approximately 5.5–5.8 in pork. However, sex-dependent hormonal influences, particularly the reduction in testicular steroids in castrated males, modulate glycogen storage and glycolytic rates in muscle, thereby impacting pH outcomes differently across treatment groups [34]. The weight-dependent reversal of pH in uncastrated females may indicate altered energy metabolism and muscle fibre type composition with increasing fatness, affecting glycolytic potential in distinct muscles. Additionally, pre-slaughter stress and handling can accelerate glycogen depletion, affecting pH decline patterns variably among immunocastrated, surgically castrated, and uncastrated pigs. The muscle-specific differences in ultimate pH, especially the higher pH seen in the ham of uncastrated females, may be explained by intrinsic metabolic and fibre-type characteristics, which influence glycogen use post-mortem. Further studies are needed to clarify the mechanistic basis of these sex- and muscle-specific pH differences.

The greater backfat thickness observed in surgically castrated males aligns with the known physiological effect of early-life androgen deprivation leading to increased lipid deposition. In contrast, the later onset of hormonal suppression in immunocastrated pigs results in intermediate fat accumulation, allowing producers to balance carcass leanness with animal welfare benefits. This dynamic is critical for optimizing carcass composition to meet market and processing demands. Multiple studies have shown that surgical castration results in increased fat deposition, aligning with the current observations [18,36]. In contrast, immunocastrated pigs generally exhibit intermediate backfat thickness, which is often less than that observed in surgically castrated males but more than in entire males [22,37]. This is likely due to the different dynamics of hormone suppression between surgical and vaccine-based methods, with surgical castration leading to altered lipid metabolism from early life and immunocastration inducing changes only after the second vaccine dose, typically administered close to slaughter.

Marbling, or IMF distribution within muscle fibres, is a critical quality attribute influencing meat flavour, juiciness, and tenderness [38,39]. In this study, marbling scores did not differ between groups with different castration methods and slaughter weights. Similarly, Martinez-Macipe et al. [40] reported similar marbling scores in surgically castrated, immunocastrated, and uncastrated Iberian pigs reared in free-ranging conditions. In contrast, Zomeno et al. [41] reported higher intramuscular fat content in surgically castrated males compared to female and entire male pigs. The relative stability of marbling across treatments in our study suggests that both castration methods produce pork with acceptable IMF levels for consumer preferences and processing requirements. However, subtle marbling differences may influence processing suitability, especially for dry-cured or specialty products where fat distribution and firmness affect texture and flavour development [42]. Therefore, while marbling was not a major differentiator in this study, monitoring IMF remains important in production systems, optimizing meat quality. In future research, evaluating detailed lipid profiles and sensory testing could increase understanding of how castration approach and slaughter weight influence not just marbling quantity but also fat quality and its role in consumer perception and product processing [43].

The fat content was significantly higher in surgically castrated male pigs at the higher slaughter weight compared to immunocastrated males, which exhibited the lowest fat levels among the groups in our study. Uncastrated and immunocastrated females showed intermediate fat values that did not differ significantly from either male group. This pattern aligns with the known effects of surgical castration on lipid metabolism, which promotes increased fat deposition [18]. In contrast, immunocastration suppresses gonadal hormones closer to the time of slaughter, resulting in a moderation of fat accumulation [22]. These differences in fat content are important as they can influence meat juiciness, flavour, and processing characteristics, with surgically castrated males tending to produce fattier carcasses under similar conditions [22].

pH is a critical indicator of meat quality, affecting tenderness, WHC, and shelf life. The ultimate pH of meat typically ranges from 5.4 to 5.8, with deviations from this range potentially indicating quality issues. Overall, the pH values observed in both lighter and heavier pigs were within the normal range for pork, with 24–48 h post-mortem pH generally between 5.5 and 5.9. Surgically castrated males and uncastrated females exhibited slightly higher pH values approaching 6.0, which may indicate a mild tendency toward darker, firmer meat, but overall meat quality remained acceptable. Previous research indicated that surgical castration and immunocastration can lead to different pH levels in meat. For instance, immunocastrated pigs have been reported to have lower ultimate pH in their loins and hams compared to surgically castrated pigs [44]. This reduction in pH could be due to the hormonal changes induced by immunocastration, which may affect muscle metabolism post-slaughter. On the other hand, entire males often exhibit higher pH values due to the higher stress and aggressive behaviour [45]. Therefore, immunocastration could be considered a method to achieve optimal pH levels without the ethical concerns associated with surgical castration.

The relationship between ultimate pH and WHC is well-established, with a higher ultimate pH generally correlating with reduced drip loss [46]. In this study, the significantly higher ultimate pH in lighter surgically castrated males and uncastrated females would theoretically predict superior WHC in these groups. While the mean values for WHC followed this expected trend, the differences were not statistically significant. This indicates that, while a strong pH effect is a crucial factor, its impact on drip loss can be modulated by other intrinsic factors related to sex and immunization status, meriting further investigation.

Moisture loss after 48 h did not differ significantly between groups. While many studies have reported increased drip and cooking loss in immunocastrated pigs compared to surgically castrated ones [47], our findings in lighter-weight groups contrast with this general pattern. Janjic et al. [48] observed that chilling loss was highest in entire males, intermediate in immunocastrates, and lowest in surgically castrated pigs, highlighting variation among castration methods and weight categories. A recent comprehensive review and meta-analysis by Škrlep et al. [22] summarized WHC, pH, color, and processing implications, concluding that surgical castrates usually exhibit the highest WHC, whereas both immunocastrates and entire males tend to show reduced WHC, which can manifest as increased drip or cooking loss in some contexts. Importantly, Škrlep et al. [22] noted that, while lower WHC can negatively affect juiciness and fresh meat processing yields, it may be advantageous for certain processed meat products, such as sausages, where reduced exudate improves formulation consistency and product stability. These nuances underscore the complexity of meat quality traits as influenced by castration method, animal weight, and intended product use.

Other quality attributes, including loin area, weight, colour, marbling, and water-holding capacity, generally did not show pronounced or statistically significant differences across treatment groups. Loin area and weight may be slightly improved in immunocastrated animals, and intramuscular fat is often reduced, but the practical impact is limited [15,21]. Meat colour variations among groups usually reflect differences in pH and fat content, which are affected by both genetic background and slaughter weight rather than castration method alone [21,34]. In the present study, uncastrated female pigs exhibited higher colour scores.

The observed differences in proximate meat composition between treatment groups in lighter and heavier pigs align well with existing studies on the effects of castration and sex on meat nutrient profiles. The significant variation in protein content among lighter pigs, where immunocastrated males and uncastrated females showed higher protein percentages compared to surgically castrated males, likely reflects the metabolic and hormonal influences of castration on muscle tissue deposition. Surgically castrated males typically exhibit reduced anabolic hormone levels, which may moderate protein accretion compared to immunocastrated males and females who retain higher natural hormone activity during growth, resulting in relatively greater protein content in their meat [8].

The finding that a higher slaughter weight did not yield statistically significant increases in backfat thickness across most treatment groups, including surgically castrated males, where a numerical difference was observed, can be explained by several factors. First, pigs do not grow linearly. As they approach physiological maturity, the composition of gain shifts towards lipid deposition, though this shift can be proportional rather than disproportionate if the animals are already in a high-fat deposition phase at the lower weight [37]. This effect is heavily modulated by genetics, as modern breeding programs have intensely selected for leanness and efficient feed conversion, inherently limiting excessive fat accumulation even at higher slaughter weights [49]. Furthermore, the specific impact of slaughter weight is consistently shown to be dependent on sex and castration status, often resulting in significant interactions rather than main effects, which aligns with the treatment-specific results observed here [34]. Finally, the high degree of individual variation in traits like backfat thickness, combined with the sample size constraints of a multi-factorial study, reduces the statistical power to detect significant differences, meaning that biologically relevant numerical trends may not achieve statistical significance [50].

The observed significant increases in longissimus dorsi cross-sectional area in immunocastrated and uncastrated females, along with the darker meat colour in heavier immunocastrated males, suggest potential quality advantages that could enhance market value. Additionally, the reduction in chilling loss and increased carcass weights in surgically castrated males at higher slaughter weights indicate improved yield efficiency. These traits imply favourable cost–benefit outcomes, where modest enhancements in carcass and meat quality parameters at increased weights could translate into better economic returns for producers without negatively impacting proximate meat composition, which remained stable across treatments [21,22].

A key limitation of this study is the absence of direct measurement of boar taint compounds. However, this was mitigated by the well-established knowledge that females and surgically castrated pigs have a low likelihood of boar taint due to the lack of testicular steroids and minimal skatole presence in adipose tissue. While sample size and grouping could limit detection of subtle effects, the multifactorial design provides a robust overview of treatment effects across relevant commercial conditions. Meat quality traits were assessed visually by trained panellists using standardised scales to ensure consistency. However, reliance on visual scoring introduces inherent subjectivity. Future studies could incorporate instrumental methods, such as colourimetry or image analysis, to enable more objective assessments. Additionally, the absence of sensory data limits the conclusion regarding consumer perception. Subsequent work should include consumer acceptance trials to complement instrumental and panel-based evaluations. The focus on two slaughter weight categories, although not exhaustive, effectively captures critical production stages, aligning with typical commercial practices. Further studies are warranted to explore the physiological mechanisms underlying minor differences in pH, fat, and protein deposition, evaluate sensory implications, and assess the practical significance of immunocastration in diverse production systems.

## 5. Conclusions

Our study confirmed that immunocastration is a viable, welfare-friendly alternative to surgical castration in pig production. While castration method and slaughter weight influenced carcass and meat quality traits, differences were generally minor. Immunocastration led to similar or improved growth performance and carcass characteristics compared with surgical castration and had only minor effects on meat quality parameters, including pH, protein content, and fat deposition. Importantly, immunocastration avoids the acute pain and welfare concerns associated with surgical castration, providing an ethically preferable option without compromising production efficiency or pork quality. These findings support immunocastration as a practical and effective strategy to balance animal welfare with industry production goals. Further research is needed to elucidate long-term impacts and to optimize immunocastration protocols across diverse production systems.

## Figures and Tables

**Table 1:** Composition of feed rations for pigs at different growth stages (g/kg).

Ingredient/Product	Grower 30–60 kg	Finisher 60–90 kg	Finisher 90–130 kg
Wheat	218.87	303.47	478.00
Maize (corn)	495.00	450.00	300.00
Wheat bran	–	–	22.05
Sunflower meal	70.00	80.00	90.00
Soybean meal	163.90	126.73	71.80
Ground limestone	8.99	9.68	11.04
Salt (grade 1)	5.35	5.07	4.86
Hydrated soybean oil	13.43	10.87	8.97
Tiamulin	0.10	–	–
Monocalcium phosphate	5.98	2.75	1.11
Methionine	1.39	0.63	0.30
Lysine (98–99%)	4.89	3.76	4.09
Tryptophan	0.23	0.10	–
Threonine (98.5–99%)	1.99	1.44	1.38
Valine	0.38	1.50	1.00
Ca Plus	2.00	1.50	0.80
Betaine Anhydrous 96%	–	–	–
7333 Additive for piglets/sows AminoPig 25	1.00	1.00	1.00
Feed additive Absorben	1.00	0.50	1.00
Amix Vet STS 150 mg	3.00	–	–
Premix for pigs (finisher 0.25%) PVT Hlobyne P 80-130 0.25/01 (Tandem)	–	2.50	2.50
Premix for pigs (grower 0.25%) PVT Hlobyne P 25-80 0.25% (Tandem)	2.50	–	–

**Table 2:** Weight-related variables measured in pigs of lighter weight.

Group	Live Weight Shrink, kg	Hot Carcass, kg	Chilled Carcass, kg	Hind Third, kg
Surgically castrated male pigs	5.2 ^a^ ± 0.09	81.5 ± 0.69	79.9 ± 0.70	13.2 ^a^ ± 0.23
Immunocastrated male pigs	5.8 ^b^ ± 0.09	82.5 ± 0.67	80.6 ± 0.68	14.0 ^ab^ ± 0.22
Uncastrated female pigs	4.8 ^c^ ± 0.09	81.6 ± 0.69	80.3 ± 0.70	14.4 ^b^ ± 0.23
Immunocastrated female pigs	4.7 ^c^ ± 0.09	81.9 ± 0.67	80.1 ± 0.68	14.6 ^b^ ± 0.22
Overall *p*-value	<0.001	0.348	0.440	0.001

Values within a column followed by different superscript letters differ significantly (*p* < 0.05).

**Table 3:** Weight-related variables measured in pigs of higher weight.

Group	Live Weight Shrink, kg	Hot Carcass, kg	Chilled Carcass, kg	Hind Third, kg
Surgically castrated male pigs	5.1 ± 0.13	100.3 ^a^ ± 0.59	98.8 ^a^ ± 0.60	15.2 ^a^ ± 0.21
Immunocastrated male pigs	5.4 ± 0.12	96.3 ^b^ ± 0.56	94.0 ^c^ ± 0.57	16.0 ^b^ ± 0.20
Uncastrated female pigs	5.0 ± 0.12	98.0 ^b^ ± 0.56	96.4 ^b^ ± 0.57	16.7 ^c^ ± 0.20
Immunocastrated female pigs	5.2 ± 0.12	98.5 ^b^ ± 0.56	96.5 ^b^ ± 0.57	15.6 ^ab^ ± 0.20
Overall *p*-value	0.086	<0.001	<0.001	<0.001

Values within a column followed by different superscript letters differ significantly (*p* < 0.05).

**Table 4:** pH measurements in pigs per live weight category.

Group	45 min Post-Mortem	24 h Post-Mortem	48 h Post-Mortem	6 Days Post-Mortem	Ham Muscle
	Pigs of lighter weight				
Surgically castrated male pigs	6.1 ± 0.06	5.7 ± 0.09	5.9 ^a^ ± 0.04	5.6 ± 0.02	5.3 ^a^ ± 0.06
Immunocastrated male pigs	6.1 ± 0.06	5.7 ± 0.08	5.5 ^b^ ± 0.04	5.5 ± 0.02	5.4 ^a^ ± 0.06
Uncastrated female pigs	6.2 ± 0.06	5.8 ± 0.09	5.8 ^a^ ± 0.04	5.6 ± 0.02	5.8 ^b^ ± 0.06
Immunocastrated female pigs	6.2 ± 0.06	6.0 ± 0.08	5.6 ^b^ ± 0.04	5.6 ± 0.02	5.5 ^ab^ ± 0.06
Overall *p*-value	0.479	0.094	<0.001	0.202	<0.001
	Pigs of higher weight				
Surgically castrated male pigs	6.3 ± 0.10	5.7 ± 0.09	5.8 ^b^ ± 0.05	5.6 ^a^ ± 0.04	5.5 ^a^ ± 0.11
Immunocastrated male pigs	6.0 ± 0.09	5.8 ± 0.08	5.5 ^a^ ± 0.05	5.4 ^b^ ± 0.04	5.6 ^a^ ± 0.10
Uncastrated female pigs	6.2 ± 0.09	5.9 ± 0.08	5.5 ^a^ ± 0.05	5.5 ^ab^ ± 0.04	6.0 ^b^ ± 0.10
Immunocastrated female pigs	6.2 ± 0.09	5.8 ± 0.08	5.6 ^a^ ± 0.05	5.6 ^ab^ ± 0.04	5.7 ^a^ ± 0.10
Overall *p*-value	0.319	0.733	<0.001	0.024	0.004

Values within a column followed by different superscript letters differ significantly (*p* < 0.05).

**Table 5:** Meat quality traits in pigs per live weight category.

Group	Backfat Thickness, mm	Longissimus Dorsi Cross-sectional Area, cm^2^	Weight of Loin, kg	Colour Score (1 = Pale, 6 = Dark)	Marbling (Scale 1–5)	Water Holding Capacity, WHC, %	Moisture Loss After 48 h, %
	Pigs of lighter weight						
Surgically castrated male pigs	22.9 ± 1.25	65.8 ± 2.17	3.1 ± 0.09	2.2 ^a^ ± 0.08	2.3 ± 0.18	61 ± 0.7	5.9 ± 0.04
Immunocastrated male pigs	20.5 ± 1.21	63.2 ± 2.10	2.9 ± 0.08	2.1 ^a^ ± 0.08	1.8 ± 0.18	61 ± 0.7	5.5± 0.04
Uncastrated female pigs	23.3 ± 1.25	67.7 ± 2.17	3.1 ± 0.09	2.5 ^b^ ± 0.08	2.0 ± 0.18	61 ± 0.7	5.8 ± 0.04
Immunocastrated female pigs	21.4 ± 1.21	67.1 ± 2.10	3.2 ± 0.08	2.4 ^ab^ ± 0.08	2.2 ± 0.18	62 ± 0.7	5.6 ± 0.04
Overall *p*-value	0.302	0.590	0.164	0.012	0.319	0.369	0.775
	Pigs of higher weight						
Surgically castrated male pigs	26.8 ^a^± 0.75	75.3 ± 2.66	3.3 ± 0.09	2.6 ± 0.09	2.4 ± 0.19	61 ± 0.6	2.1 ± 0.29
Immunocastrated male pigs	23.6 ^b^ ± 0.71	72.2 ± 2.51	3.4 ± 0.08	2.3 ± 0.08	1.9 ± 0.18	61 ± 0.6	2.7 ± 0.27
Uncastrated female pigs	25.7 ^ab^ ± 0.71	75.3 ± 2.51	3.6 ± 0.08	2.4 ± 0.08	2.1 ± 0.18	61 ± 0.6	1.8 ± 0.27
Immunocastrated female pigs	25.0 ^ab^ ± 0.71	72.9 ± 2.52	3.3 ± 0.08	2.4 ± 0.08	2.5 ± 0.18	62 ± 0.6	2.6 ± 0.27
Overall *p*-value	0.028	0.749	0.094	0.176	0.101	0.516	0.061

Values within a column followed by different superscript letters differ significantly (*p* < 0.05).

**Table 6:** Proximate composition of meat from pigs per live weight category.

Group	Protein, %	Fat, %	Moisture, %	Collagen, %
	Pigs of lighter weight			
Surgically castrated male pigs	23.0 ^a^ ± 0.14	2.2 ± 0.17	73.7 ± 0.16	0.64 ± 0.03
Immunocastrated male pigs	23.5 ^b^ ± 0.13	1.9 ± 0.16	73.5 ± 0.15	0.72 ± 0.03
Uncastrated female pigs	23.5 ^b^ ± 0.14	2.0 ± 0.17	73.4 ± 0.16	0.71 ± 0.03
Immunocastrated female pigs	23.4 ^ab^ ± 0.14	2.0 ± 0.16	73.5 ± 0.15	0.73 ± 0.03
Overall *p*-value	0.047	0.749	0.658	0.155
	Pigs of higher weight			
Surgically castrated male pigs	23.2 ± 0.12	2.7 ^b^ ± 0.17	73.9 ± 0.29	0.69 ± 0.03
Immunocastrated male pigs	23.5 ± 0.11	2.0 ^a^ ± 0.16	73.3 ± 0.27	0.73 ± 0.03
Uncastrated female pigs	23.3 ± 0.11	2.2 ^ab^ ± 0.16	73.4 ± 0.27	0.79 ± 0.03
Immunocastrated female pigs	23.4 ± 0.11	2.2 ^ab^ ± 0.16	73.2 ± 0.27	0.77 ± 0.03
Overall *p*-value	0.292	0.036	0.299	0.054

Values within a column followed by different superscript letters differ significantly (*p* < 0.05).

**Table 7:** Statistical significance (*p*-value) of the effect of slaughter weight (lighter vs. heavier) within each sex and castration treatment group.

Group	Surgically Castrated Male Pigs	Immunocastrated Male Pigs	Uncastrated Female Pigs	Immunocastrated Female Pigs
pH values				
45 min post-mortem	0.592	0.812	1.000	1.000
24 h post-mortem	0.997	1.000	1.000	0.724
48 h post-mortem	0.993	**<0.001**	**<0.001**	0.509
6 days post-mortem	1.000	0.849	0.674	1.000
Ham muscle	0.919	0.999	**<0.001**	0.333
Meat quality traits				
Backfat thickness, mm	0.236	0.337	0.841	0.988
Longissimus dorsi cross-sectional area, cm^2^	0.113	**0.019**	**0.013**	0.091
Weight of loin, kg	0.401	**0.010**	**<0.001**	**0.009**
Colour (scale 1–5)	0.121	**0.005**	0.999	1.000
Marbling	1.000	0.999	0.996	1.000
WHC, %	1.000	0.999	1.000	1.000
Moisture loss after 48 h, %	1.000	0.908	0.992	1.000
Proximate composition of meat				
Protein, %	0.960	1.000	0.971	1.000
Fat, %	0.497	1.000	0.976	0.996
Moisture, %	0.992	1.000	1.000	1.000
Collagen, %	0.796	0.999	0.347	0.850

*p*-values are from Tukey–Kramer pairwise comparisons of least squares means within each row (treatment group). Significant differences (*p* < 0.05) are highlighted in bold.

## Data Availability

The data presented in this study are available upon request from the corresponding author.

## Abbreviations

The following abbreviations are used in this manuscript:

IMF	Intramuscular fat
WHC	Water-holding capacity
